# Persistent Cannabis Abuse and Risk for Hospitalization for Acute Pancreatitis: A Cross-Sectional Study in United States Hospitals

**DOI:** 10.7759/cureus.15601

**Published:** 2021-06-11

**Authors:** Renu Bhandari, Siddharth Gupta, Karnav Modi, Maharshi R Raval, Hajara Joundi, Jeet R Patel, Amanpreet K Pannu, Prerna Sharma

**Affiliations:** 1 Medicine, Manipal College of Medical Sciences, Kaski, NPL; 2 Internal Medicine, Sri Guru Ram Das Institute of Medical Sciences and Research, Amritsar, IND; 3 Internal Medicine, Byramjee Jeejeebhoy (BJ) Medical College, Ahmedabad, IND; 4 Internal Medicine, University Cadi Ayyad, Faculty of Medicine and Pharmacy, Marrakech, MAR; 5 Internal Medicine: Pediatrics, Byramjee Jeejeebhoy (BJ) Medical College, Ahmedabad, IND; 6 Medicine, Sri Guru Ram Das University of Health Sciences, Amritsar, IND; 7 Psychiatry, Government Medical College, Amritsar, IND

**Keywords:** acute pancreatitis, cannabis use disorder, cannabis research, marijuana abuse, national inpatient sample, risk-factors

## Abstract

Objectives

To explore the independent association between cannabis abuse and subsequent hospitalizations for acute pancreatitis (AP) and delineate the demographic differences among AP in patients with and without persistent cannabis abuse.

Methods

We conducted a retrospective cross-sectional study using the nationwide inpatient sample and included 50,444,133 patients (age 18-50 years) with a primary discharge diagnosis for medical illnesses and further grouped by presence of AP (N = 666,248). We used the logistic regression model to measure the odds ratio (OR) of the association between cannabis abuse and hospitalization for AP and adjusted it for demographic confounders and comorbid risk factors.

Results

Cannabis abuse significantly increases the odds for AP-related hospitalization (OR 2.12, P <0.001). When the regression model was controlled for potential risk factors (gall stones, cystic fibrosis, hypertriglyceridemia, hypercalcemia, hyperparathyroidism, abdominal surgeries, tobacco abuse, and alcohol abuse), cannabis abuse did not increase the odds for AP-related hospitalization (OR 0.72, P <0.001) due to the significant effect caused by gallstones (OR 30.98, P <0.001) and alcohol abuse (OR 12.69, P <0.001). AP inpatients with cannabis abuse were younger compared to non-cannabis abusers (mean age, 35.7 vs. 37.9 years), and majorly male (70.9% vs. 53.8%). AP was considerably more prevalent in whites (60.6%), followed by blacks (18.3%) and Hispanics (15.2%).

Conclusion

Cannabis abuse increased the unadjusted odds for AP-related hospitalization by two times, but after controlling for potential risk factors the adjusted odds of association significantly reduced. Cannabis-induced AP can be treated if a problematic recreational cannabis use pattern is discontinued at an earlier stage. Therefore, awareness campaigns and early supportive therapy among cannabis abusers might help diagnose and treat the comorbidity and improve the quality of life.

## Introduction

Acute pancreatitis (AP) is a severe inflammatory condition associated with significant morbidity and mortality. It is responsible for about 275,000 hospital admissions every year in the United States (US), costing more than 2.5 billion dollars annually [1]. Overall, the incidence is 34 per 100,000 general population per year and affects men and women equally. The incidence of AP is increasing and compared to the European and Western Pacific regions, the incidence and mortality are significantly higher in the US [2-3].

In the US, cannabis is the most commonly used illicit substance, and its use has increased from 8.2% to 14.5% of the total US population from 1992 to 2016 [4]. Synthetic cannabinoids are more common in younger users, as they are inexpensive and easily accessible. In addition, they have much higher efficacy and affinity to bind to cannabinoid receptors as compared to the natural forms. Their appeal is further enhanced to the younger demographic, as they are also challenging to identify with routine drug screenings [5].

According to the substance abuse and mental health services administration, cannabis is the most commonly abused drug among those who drink, with the exception of tobacco. Among current drinkers, 11.4% reported cannabis use in the past 12 months; 3.9% reported concurrent cannabis use (concurrent users) and 7.5% of drinkers reported they usually or sometimes used cannabis with alcohol (simultaneous users) [6].

The relationship between cannabis and pancreatitis has always been a topic of debate, as there is limited literature. Some studies report cannabis causing pancreatitis while other studies support the protective effect of cannabis in alcohol-induced pancreatitis [7]. Approximately 20% of AP cases are classified as idiopathic. The diagnosis of cannabis-associated pancreatitis is made after an extensive workup fails to identify any other cause. This accounts for a small fraction of idiopathic cases of AP. Cannabis use is associated with nine percent of idiopathic cases and ten percent of all first episodes of AP [8]. So, we conducted an inpatient study to explore the independent association of cannabis abuse and hospitalizations for AP after controlling for potential risk factors and demographic confounders. Furthermore, we delineated the demographic differences among AP in patients with versus without cannabis abuse.

## Materials and methods

We conducted a retrospective cross-sectional study using the Nationwide Inpatient Sample (NIS, 2010 to 2014), the largest database of hospital stays in the US. It is a reliable data source for epidemiological estimates that involve the risk factors associated with diseases [9]. We included 50,444,133 patients (age 18-50 years) with a primary discharge diagnosis for medical conditions and were further grouped by the presence of AP (N = 666,248).

The demographic variables studied in this analysis are age, sex, and race [10]. The existing risk factors for AP were defined based on current literature and included gall stones, cystic fibrosis, hypertriglyceridemia, hypercalcemia, hyperparathyroidism, abdominal surgeries, tobacco abuse, alcohol abuse, and cannabis abuse.

We compared the non-AP and AP groups using bivariate analysis using descriptive statistics to delineate the differences in demographics and comorbid risk factors. The first model of logistic regression analyses was conducted to determine the odds ratio (OR) of association between cannabis abuse and hospitalization for AP, and in the next model, it was adjusted for demographics and comorbid risk factors (gall stones, cystic fibrosis, hypertriglyceridemia, hypercalcemia, hyperparathyroidism, abdominal surgeries, tobacco abuse, and alcohol abuse). Next, we conducted the bivariate analysis using descriptive statistics and the *P* values were generated using the Pearson's chi-square test to compare the demographics in cannabis users and non-users hospitalized for AP. All statistical analyses were performed in the statistical package for the social sciences (SPSS) version 26.0 (IBM Corp., Armonk, NY) with a statistical significance set a priori at* P-value* <0.01.

Unique patient identifiers were used to protect the patient identity. So the use of the NIS, according to the US department of health and human services, does not require approval from an institutional review board [9].

## Results

The prevalence of AP in the medical inpatient population was 1.32% (666,248 out of 50,444,133) and were older than non-AP inpatients (mean age, 37.8 vs. 33.9 years). Males had higher odds (OR 1.54; 95 CI 1.537 - 1.555) compared to females for being hospitalized for AP. AP was considerably more prevalent in whites (60.6%), followed by blacks (18.3%) and Hispanics (15.2%).

In our study, there exists a statistically significant association between certain risk factors and AP. Patients with gallstones had the highest odds of AP hospitalization (OR 30.98; 95% CI 30.699 - 31.253), followed by alcohol abuse (OR 12.69; 95% CI 12.613 - 12.776), cystic fibrosis (OR 2.55; 95% CI 2.417 - 2.697), hypertriglyceridemia (OR 2.53; 95% CI 2.507 - 2.542), and hypercalcemia (OR 2.26; 95% CI 2.180 - 2.340). The previous history of abdominal surgery was not shown to have a statistically significant risk for AP hospitalization.

Cannabis abuse significantly increased the odds for AP hospitalization (OR 2.12; 95% CI 2.095 - 2.149). But when the regression model was adjusted for demographics and other potential risk factors, cannabis abuse did not increase the odds for AP hospitalization (OR 0.72; 95% CI 0.713 - 0.734), as shown in Table 1.

**Table 1:** Regression analysis for acute pancreatitis hospitalization *(-) refers to non-acute pancreatitis and (+) refers to acute pancreatitis groups. OR: odds ratio; CI: confidence interval; SD: standard deviation.

Variables	Acute pancreatitis		Logistic regression model		
	(-)* in %	(+)* in %	OR	95% CI	P-value
Total Patients	49777885	666,248	-	-	-
Mean age (SD), in years	33.9 (9.3)	37.8 (8.6)	1.00	1.003 – 1.004	<0.001
Gender					
Male	27.5	54.5	1.54	1.537 – 1.555	<0.001
Female	72.5	45.5	Reference		
Race					
White	56.1	60.6	Reference		
Black	19.0	18.3	0.93	0.925 – 0.938	<0.001
Hispanic	16.7	15.2	0.95	0.940 – 0.955	<0.001
Other	8.1	5.9	0.81	0.799 – 0.817	<0.001
Risk factors					
No risk factor	-	-	Reference		
Gall stones	0.8	16.0	30.98	30.699 – 31.253	<0.001
Cystic fibrosis	0.1	0.2	2.55	2.417 – 2.697	<0.001
Hypertriglyceridemia	8.0	21.1	2.53	2.507 – 2.542	<0.001
Hypercalcemia	0.2	0.6	2.26	2.180 – 2.340	<0.001
Hyperparathyroidism	0.1	0.1	1.31	1.221 – 1.406	<0.001
Abdominal surgeries	13.1	20.7	0.99	0.987 – 1.001	0.086
Tobacco abuse	14.5	37.1	1.61	1.597 – 1.617	<0.001
Alcohol abuse	3.7	38.0	12.69	12.613 – 12.776	<0.001
Cannabis abuse	1.8	3.8	0.72	0.713 – 0.734	<0.001

Approximately 3.8% (N = 25,478) of 666,248 AP inpatients were cannabis abusers. AP inpatients with cannabis abuse were younger as compared to non-cannabis abusers (mean age, 35.7 vs. 37.9 years). A higher proportion of cannabis abusers were male (70.9% vs. 53.8% in non-cannabis abusers) and blacks (32.6% vs. 17.7% in non-cannabis abusers) as shown in Table 2.

**Table 2:** Acute pancreatitis inpatients by cannabis abuse *(-) refers to non-cannabis abuse and (+) refers to cannabis abuse groups. SD: standard deviation

Variables	Cannabis abuse		P-value
	(-)* in %	(+)* in %	
Total patients	640770	25478	-
Mean age (SD), in years	37.9 (8.6)	35.7 (8.7)	<0.001
Gender			
Male	53.8	70.9	<0.001
Female	46.2	29.1	
Race			
White	61.0	50.5	<0.001
Black	17.7	32.6	
Hispanic	15.3	12.1	
Other	6.0	4.8	

## Discussion

Men in our study sample had 1.5 times higher odds for AP-related hospitalization than females. This may be due to increased comorbidities seen in men like diabetes, hypertension, and coronary artery disease; also, they are more likely to smoke cigarettes and abuse drugs [11]. Whites have a higher association of being hospitalized due to AP, whereas blacks, Hispanics, and other races/ethnicities didn't have any significant relationship. It may be because whites are more likely to consume alcohol, which is a potential risk factor for worsening symptoms of AP [12].

Among the risk factors, gallstones and alcohol abuse increased the likelihood of AP-related hospitalization. Gallstone exacerbates AP by increasing duct pressure, which leads to unregulated activation of digestive enzymes [13]. Ethanol and its metabolite produce oxidative stress or the generation of fatty acid ethyl esters, leading to inflammation, which may be the reason for causing acute pancreatitis [12]. Simultaneous usage of alcohol and cannabis is found to be nearly twice as common as concurrent use, suggesting that people who reported consuming both did so at the same time [6]. In our study, cystic fibrosis increased the risk by 2.5 times, as it alters the composition of pancreatic secretions, which ultimately leads to acinus plugging and dilation, causing epithelial injury and destruction accompanied by inflammation [14]. Also, hypertriglyceridemia and hypercalcemia had a higher impact on increasing the likelihood of AP-related hospitalization. Although the precise cause is not understood, the hypothesis may be that chylomicron and free fatty acids, which increase plasma viscosity, may trigger ischemia and organ inflammation [15]. It is hypothesized that hypercalcemia causes pancreatic damage by conversion of trypsinogen to trypsin [16].

In our study, it was found that cannabis abusers had twice the higher risk of association with increased hospitalization. After controlling for other risk factors, we didn't find an increase in hospitalization due to acute pancreatitis. A similar result was seen in the study where the patient with cannabis exposure had significantly lower inpatient mortality than the non-cannabis group [17]. Cannabinoids function by stimulating two receptors, cannabinoid receptor type 1 (cb1) and type 2 (cb2), within the human body's endocannabinoid system. Tetrahydrocannabinol binds to activate cannabinoid receptors, predominantly cb1 receptors in the pancreas [18-19]. Activation of cb1 receptors initiates a proinflammatory cascade leading to hypotension, tachycardia, altered consciousness, and septic shock [20-21]. Cerulein administration, a natural endogenous ligand for the cb1 receptor, leads to decreased blood flow and affecting deoxyribonucleic acid (DNA) synthesis leading to pancreatic edema and inflammatory infiltration [22]. Cannabinoids reduce blood flow by reducing blood vessel proangiogenic factors and vascular endothelial growth factors. Activation of cb1 or cb2 receptors can also inhibit adenylyl cyclase activity and decrease cyclic adenosine monophosphate levels, which ultimately affects gene transcription and cause apoptosis [18]. Basile et al. study showed a 1.2 times higher risk of post-endoscopic retrograde cholangiopancreatography pancreatitis in cannabis abusers due to tissue injury caused by cannabis [23]. A study by Matsuda et al. found that the downregulation of the endocannabinoid system by am251 (cannabinoid cb1 receptor inhibitor) with severe AP showed an improved survival rate in rats [24]. On the contrary, Michalski et al. study showed hu210, a synthetic agonist of cannabinoid receptors cb1 and cb2, resolved the abdominal pain induced by AP and decreased the inflammation of the pancreas, shows therapeutic benefits of the pancreas [25]. Also, a study by Barkin et al. showed cannabis could be the potential risk factor for causing AP and recurrent AP in a young population under 35 years of age [26].

The limitation of this study includes the fact that it is a cross-sectional study and, hence, cannot establish or deny any causal relationship between AP and cannabis abuse. The NIS is administrative data, lacks patient-level clinical information, and sampling was based on diagnostic codes, which may have resulted in underreporting of comorbidities and is subject to prevalence-incidence bias. So, there exists an information bias due to a lack of details on the amount, dose, frequency, and last episodic use of cannabis for the study in patients, which can affect the association/relationship with AP hospitalization. Yet, the NIS offers the largest inpatient data pool in the US. Hence, the data is incomparable to the population-based perception of disease associations with systematic and temporal factors. The sample size is very large and is diverse and represents the inpatient population across the US.

## Conclusions

Cannabis abuse does not independently increase the odds of AP-related hospitalization. Cannabis-induced AP can be treated if a persistent cannabis abuse pattern is discontinued at an earlier stage. Therefore, awareness campaigns and early interventional supportive therapies among cannabis abusers might help diagnose and treat comorbid cannabis and other substance-use disorders and improve the quality of life. Many studies concerning the pathophysiology of cannabis abuse and AP are required in both animal and human models to identify the impact and also to address and treat persistent cannabis abuse patterns on gastrointestinal health.
